# Silent Tyrosinemia Type I Without Elevated Tyrosine or Succinylacetone Associated with Liver Cirrhosis and Hepatocellular Carcinoma

**DOI:** 10.1002/humu.23047

**Published:** 2016-08-08

**Authors:** Patrick R. Blackburn, Raymond D. Hickey, Rebecca A. Nace, Nasra H. Giama, Daniel L. Kraft, Andrew J. Bordner, Roongruedee Chaiteerakij, Jennifer B. McCormick, Maja Radulovic, Rondell P. Graham, Michael S. Torbenson, Silvia Tortorelli, C. Ronald Scott, Noralane M. Lindor, Dawn S. Milliner, Devin Oglesbee, Wafa'a Al‐Qabandi, Markus Grompe, Dimitar K. Gavrilov, Mounif El‐Youssef, Karl J. Clark, Paldeep S. Atwal, Lewis R. Roberts, Eric W. Klee, Stephen C. Ekker

**Affiliations:** ^1^ Center for Individualized Medicine, Mayo ClinicRochesterMinnesota; ^2^ Palo Alto Medical FoundationMountain ViewCalifornia; ^3^ Chulalongkorn University and King Chulalongkorn Memorial HospitalBangkokPathumwan; ^4^ Department of Pediatrics, Division of Genetic MedicineUniversity of WashingtonSeattleWashington; ^5^ Department of Health Science ResearchMayo ClinicScottsdaleArizona; ^6^ Deptartment of Pediatrics, Faculty of MedicineUniversity of Kuwait24923 SafatKuwait CityKuwait; ^7^ Department of Pediatrics, Papé Family Pediatric Research Institute, Oregon Science & Health UniversityPortlandOregon; ^8^ Center for Individualized Medicine, Mayo ClinicJacksonvilleFlorida

**Keywords:** fumarylacetoacetate hydrolase, FAH, tyrosinemia type I, TYRSN1, TYR I, newborn screening, whole exome sequencing, pediatric liver cancer

## Abstract

Tyrosinemia type I (TYRSN1, TYR I) is caused by fumarylacetoacetate hydrolase (FAH) deficiency and affects approximately one in 100,000 individuals worldwide. Pathogenic variants in *FAH* cause TYRSN1, which induces cirrhosis and can progress to hepatocellular carcinoma (HCC). TYRSN1 is characterized by the production of a pathognomonic metabolite, succinylacetone (SUAC) and is included in the Recommended Uniform Screening Panel for newborns. Treatment intervention is effective if initiated within the first month of life. Here, we describe a family with three affected children who developed HCC secondary to idiopathic hepatosplenomegaly and cirrhosis during infancy. Whole exome sequencing revealed a novel homozygous missense variant in *FAH* (Chr15(GRCh38):g.80162305A>G; NM_000137.2:c.424A > G; NP_000128.1:p.R142G). This novel variant involves the catalytic pocket of the enzyme, but does not result in increased SUAC or tyrosine, making the diagnosis of TYRSN1 problematic. Testing this novel variant using a rapid, in vivo somatic mouse model showed that this variant could not rescue FAH deficiency. In this case of atypical TYRSN1, we show how reliance on SUAC as a primary diagnostic test can be misleading in some patients with this disease. Augmentation of current screening for TYRSN1 with targeted sequencing of *FAH* is warranted in cases suggestive of the disorder.

## Introduction

Tyrosinemia type I (MIM# 276700; TYRSN1, TYR I) is an inborn error of tyrosine metabolism caused by pathogenic variants in fumarylacetoacetate hydrolase (*FAH*; MIM# 613871, EC 3.7.1.2), the final enzyme in the tyrosine degradation cascade [Grompe, 2001]. It is inherited in an autosomal recessive pattern, and the estimated carrier frequency is ∼1:100 in the United States [King et al., 2006]. The disorder affects approximately one in 100,000 to 120,000 individuals worldwide [de Laet et al., 2013; King et al., 2006]. In all described cases to date, pathogenic variants in *FAH* lead to the accumulation of two toxic metabolites, fumarylacetoacetate (FAA) and succinylacetoacetate (SAA), in the liver and renal proximal tubules [Grompe, 2001]. FAA is particularly labile and reacts with thiol groups in proteins, such as glutathione, and causes DNA damage, leading to extensive cirrhosis in the liver and renal tubular acidosis in the kidney presenting as Renal Fanconi Syndrome [Jorquera and Tanguay, 2001].

Patients can have either an acute or chronic presentation, and there is no clear correlation between patient genotype and the severity of the clinical presentation [Poudrier et al., 1998]. Patients typically exhibit elevations in serum tyrosine, methionine, and phenylalanine as well as markedly increased alpha‐fetoprotein (AFP) levels [Grompe, 2001; King et al., 2006]. SAA undergoes spontaneous decarboxylation to form succinylacetone (SUAC). The detection of SUAC in the blood or urine is pathognomonic for the disorder and is the current best newborn screening test for TYRSN1, even though implementation of this assay has been inconsistent across laboratories in the United States [American College of Medical Genetics Newborn Screening Expert Group, 2006; de Laet et al., 2013]. NTBC (2‐(2‐nitro‐4‐trifluoro‐methylbenzyol)‐1,3 cyclo‐hexanedione, or nitisinone) is the current treatment for this disorder and can minimize liver and renal damage when commenced during the first few weeks of life, thereby obviating the need for a future liver transplant [Grompe, 2001].

Here, we describe a family with several affected members who have a novel homozygous variant in *FAH* (Chr15(GRCh38):g.80162305A > G; NM_000137.2:c.424A > G; NP_000128.1:p.R142G) with undetectable SUAC and normal amino acid levels but extensive cirrhosis and susceptibility to hepatocellular carcinoma (HCC). Despite having a homozygous missense variant in *FAH*, these patients lacked the characteristic biochemical abnormalities associated with TYRSN1, and a more extensive investigation was required to establish the pathogenicity of this novel variant.

## Materials and Methods

### IRB and Patient Consent

We evaluated the initial three patients, their unaffected parents, and two unaffected siblings. Family members were consented for sample collection and subsequent analysis under a protocol approved by the institutional review board of the Mayo Clinic. A separate protocol approved by the Mayo Clinic institutional review board was used to consent the proband, mother, and another male sibling for whole exome sequencing. All the patients or their parents provided written informed consent.

### ***Fah ^−^^/^^−^*** Mice and Injections

Mice of the Fah exon 5 strain (C57Bl6/6J‐Fah^−/−^ mice) were treated according to the National Institute of Health (NIH) Guide for the Care and Use of Laboratory Animals and with the approval of the Mayo Clinic Institutional Animal Care and Use Committee [Grompe et al., 1993]. Fah^−/−^ mice were given 4 mg/l NTBC in water ad libitum until the day of injection (Yecuris, Tualatin, OR). Fah^−/−^ mice that were not given NTBC progressively lost weight in the absence of therapeutic gene rescue. Plasmid DNA was diluted in Ringer's solution and was hydrodynamically injected into the tail vein for 5–8 sec, as previously described [Bell et al., 2007]. The injection volume was 10% of the weight of the animal. Animals weighing more than 25 g were given 2.5 ml of plasmid DNA in Ringer's solution. A 1:1 molar ratio of transposon to transposase vector was utilized. Twenty micrograms of the *Sleeping Beauty* transposon containing either WT or p.R142G human *FAH* cDNA (pKT2/hFAHIL or pKT2/R142G/hFAHIL) was co‐injected with 11.5 μg of the *Sleeping Beauty* transposase‐containing vector (pKUb‐SB100X) [Wangensteen et al., 2008]. Following hydrodynamic injections, NTBC was removed, and the body weight of the animals was measured weekly. Animals were imaged 24 hr post‐hydrodynamic injection and subsequently at 1‐week intervals over ∼2 months. Briefly, animals were injected intraperitoneally with 0.2 ml of 15 mg/ml d‐luciferin potassium salt (Gold Biotechnology, St. Louis MO) dissolved in phosphate‐buffered saline. After 5 min, the animals were anesthetized using 3% isofluorane inhalation and imaged from 1 sec to 3 min using the Xenogen IVIS 200 imaging system (Xenogen Corp., Alameda, CA). Luciferase activity levels (as luminescence) were expressed in total flux radiance or photons emitted per second per centimeter cubed (p/sec/cm^3^/sr).

### Western Blot Analyses

Samples were homogenized in cell lysis buffer (Cell Signaling, Danvers, MA). Total protein was separated by SDS‐PAGE and then transferred to a polyvinylidene fluoride membrane (TransBlot Turbo; BioRad, Hercules, CA). Anti‐FAH (in‐house antibody; Grompe laboratory) and anti‐beta Actin (#4970; Cell Signaling) primary antibodies were detected using a horseradish peroxidase‐conjugated anti‐rabbit secondary antibody (Cell Signaling) and imaged using a chemiluminescent substrate (Thermo Scientific, Waltham, MA) [Wang et al., 2002].

### Cell Culture

The Huh7 HCC cell line was cultured in DMEM (Thermo Fisher Scientific, Cambridge, MA) supplemented with 10% heat‐inactivated fetal bovine serum, 100 U/ml penicillin, and 100 μg/ml streptomycin. The media was changed every 2 to 3 days, and cells were passaged using 0.05% Trypsin/ethylenediaminetetraacetic acid (Thermo Fisher Scientific).

### FAH Enzyme Kinetic Tests

The assay is based on the disappearance of absorbance at 330 nm [Knox and Edwards, 1955]. FAA has a molar extinction coefficient of 13,500 M^−1^ cm^−1^ at 330 nm [Knox and Edwards, 1955]. Huh7 cells were trypsinized with 0.05% Trypsin/ethylenediaminetetraacetic acid, harvested and pelleted by centrifugation. Cell pellets were resuspended in 200 μl of 0.25 M of ice‐cold sucrose solution. The samples were vortexed briefly to resuspend the pellets and then sonicated (Microson Ultrasonic Cell Disruptor) on ice twice for 10 sec at full amplitude (output power setting of 20 Watts). The samples were the centrifuged for 10 min at 14,000*g* at 4°C. The supernatants were transferred to fresh tubes and placed on ice. The enzyme kinetic assay was conducted using a Nanodrop 2000c spectrophotometer (Thermo Scientific). Phosphate‐buffered saline (570 μl) and 20 μl of protein extract were added to a glass cuvette and the sample was blanked. Ten microliters of FAA (optical density: ∼1 = 7.5 × 10^−5^ M) was then added to the cuvette, and the sample was mixed by inversion. The cuvette was placed back in the spectrophotometer, and the absorbance was measured at 330 nm at 15 sec intervals for 5 min at room temperature.

### Statistical Analysis

Unless otherwise stated, an unpaired two‐sided Student's *t*‐test was used to assess statistical significance. Significant findings had a *P* ≤ 0.05.

## Results

### Case Description

**Figure 1:**
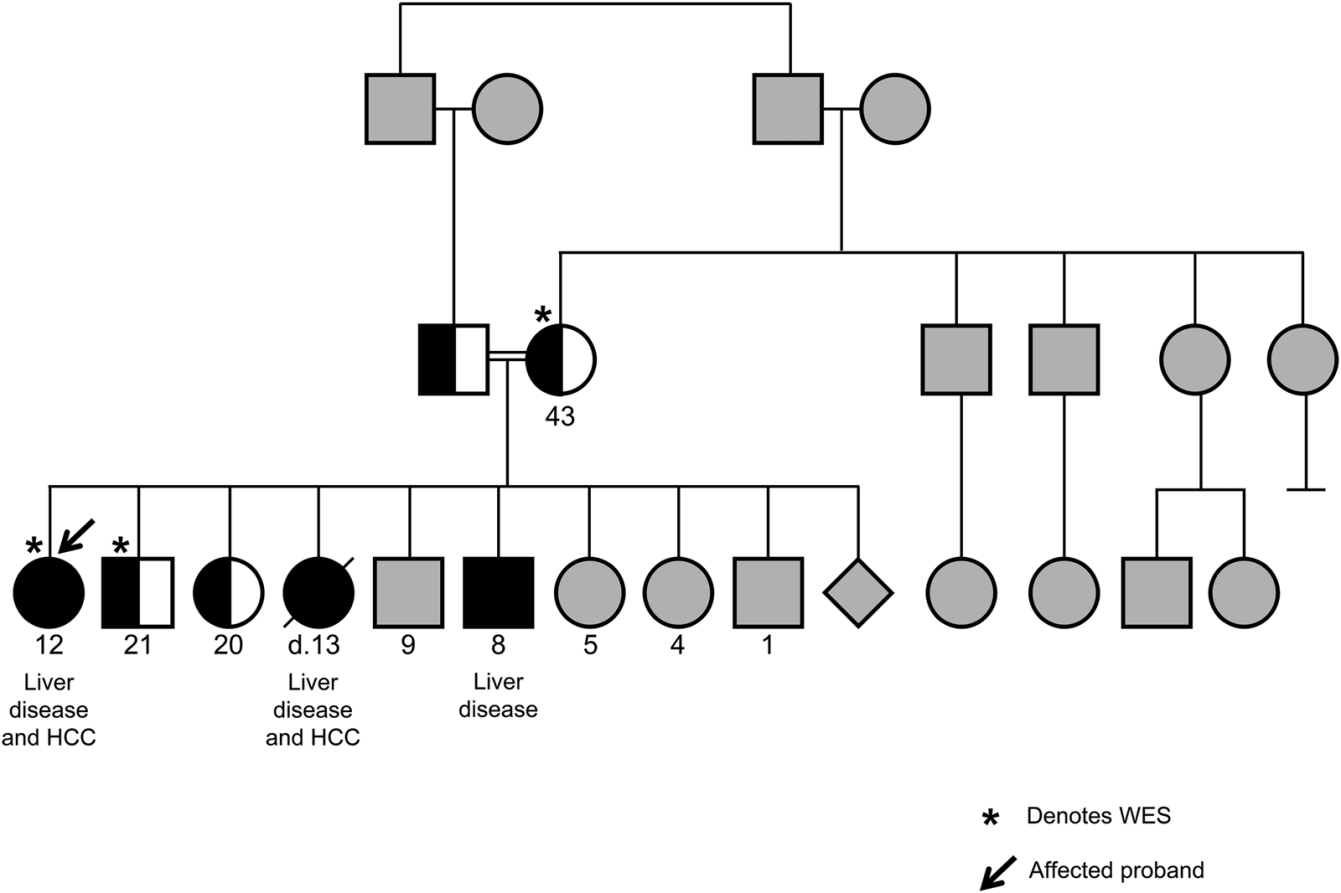
Pedigree of the affected family with hepatocellular carcinoma showing FAH variant status. Whole exome sequencing was done on three family members (denoted with *): the proband (black arrow), unaffected sibling, and mother. Both parents were carriers for the p.R142G variant in the FAH gene. Two other children in this family were sequenced and found to be heterozygous carriers for this variant. The three children with liver disease had the p.R142G homozygous variation in FAH. Gray shading indicates that variant status was not determined.

Two female patients, born to consanguineous parents, were each diagnosed at 12 and 13 years of age as having HCC (Fig. 1). Both children had a history of hepatosplenomegaly, abdominal bloating, and vomiting beginning in the first few months of life. A liver biopsy of the proband at about 4 months of age revealed fibrosis and increased glycogen storage; consequently, the children received a tentative diagnosis of glycogen storage disorder type III. Both children were monitored for several years with no increase in liver size or abnormal laboratory findings. At the age of 13 years, the proband's sister was admitted to the hospital with cachexia and abdominal pain. A CT scan showed hepatomegaly and a focal liver lesion that was found to be HCC with associated vascular invasion. The child's AFP levels were >40,000 ng/ml (normal range < 6 ng/ml; Table 1). The child was referred for specialized care but died before she could be evaluated.

**Table 1:** Clinical Laboratory Values for Affected Individuals with the Homozygous p.R142G *FAH* Variant

	Proband	Sister	Brother	Reference range
**Blood**				
AFP	**32,936**	**>40,000**	**41**	<6 ng/ml
GGTP	14	–	18	9–24 U/l
AST	21	–	21	8–60 U/l
ALT	12	–	14	7–55 U/l
Bilirubin, Total, S	0.4	–	0.3	0.0–0.3 mg/dl
Bilirubin, Direct	0.1	–	0.1	0.0–0.3 mg/dl
PT	12.4	–	12	9.5–13.8 sec
APTT(S)	36	–		28–38 sec
INR	1	–	1	0.8–1.2
Tyrosine	55	–	106	31–106 nmol/ml
Phenylalanine	61	–	70	30–95 nmol/ml
Methionine	28	–	35	11–37 nmol/ml
ALA dehydratase	7.0	–	4.1	No reference range
**Urine**				
5‐Aminolevulinic acid	16	–	**25**	≤20 nmol/ml
Organic acids	None detected	–	None detected	No reference range
Tyrosine	71	–	**212**	12–208 nmol/mg Cr
Methonine	8	–	18	<20 nmol/mg Cr
Phenylalanine	80	–	110	11–111 nmol/mg Cr

Clinical laboratory values for the proband, older deceased sister, and the affected younger brother. No abnormal urine organic acids (including succinylacetone, SUAC) were detected. Values in **bold** are outside the reference range.

**Figure 2:**
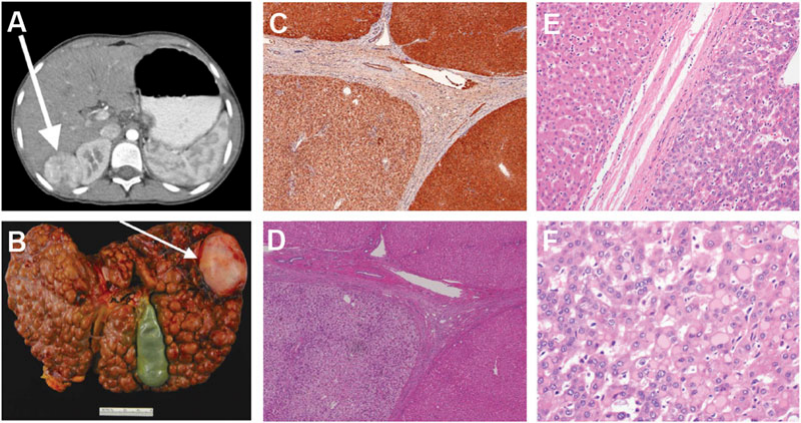
Imaging, pathological, and immunohistological study findings on the proband. **A**: CT scan showing the 4.8 × 3.6 × 3.6 cm^3^ enhancing mass in segment VI of the proband's liver. Biopsy showed moderately differentiated hepatocellular carcinoma. **B**: Explanted liver from the proband showing extensive macronodular cirrhosis. The arrow points to the hepatocellular carcinoma lesion shown in the CT scan in panel A. **C**: Fumarylacetoacetate hydrolase immunohistochemistry of liver tissue derived from the proband (10×). Macronodules were immunopositive for fumarylacetoacetate hydrolase with some variability in staining. **D**: Hematoxylin and eosin stain of liver tissue taken from the proband (10×). The nonneoplastic liver parenchyma showed inactive cirrhosis (stage 4) that was morphologically cryptogenic. **E**: Hematoxylin and eosin stained section at the tumor margin (20×). **F**: Hematoxylin and eosin stained section showing multiple changes in clear cell foci and mild dysplasia (40×). Pale bodies were also present.

Shortly after her sister's death, the proband was evaluated by CT scan and ultrasound imaging and was found to have a focal lesion (5 × 4 × 3 cm^3^) in segment IV of the liver (Fig. 2A). At this time, she was referred to the Mayo Clinic (Rochester, MN) for transplant evaluation. A repeat abdominal CT scan confirmed that she had a liver mass and partial calcification of her kidneys. The patient was evaluated for TYRSN1 and her urine organic acid levels were normal on two separate occasions without detectable SUAC (Table 1). No other abnormal metabolites were detected. Additionally, her urine and plasma amino acids were within the normal ranges. Glycogen storage disorders were excluded by biochemical testing. Given the rapid progression of HCC after diagnosis in her sibling, she was treated with transarterial chemoembolization followed by liver transplantation that was curative.

### Liver Pathology

The explanted liver showed extensive micro‐ and macronodular cirrhosis (Fig. 2B). Histopathologic examination of the explanted liver revealed a necrotic 4.6‐cm mass in the right lobe of the liver that was consistent with prior chemoembolization. Adjacent to this were two viable satellite nodules measuring 0.8 and 0.7 cm in greatest dimension. These nodules featured a proliferation of hepatocytes forming thickened trabecula with unpaired arteries, which is characteristic of HCC. Intracytoplasmic pale bodies were identified, but lamellar fibrosis was absent (Fig. 2F). The non‐neoplastic liver showed microvesicular steatosis involving 40% of the parenchyma and was associated with established cirrhosis, which was morphologically cryptogenic (Fig. 2C–E). There were multiple foci of clear cell changes within the cirrhotic nodules (Fig. 2E–F). Periodic Acid–Schiff and Periodic Acid–Schiff with diastase stains excluded glycogen accumulation as the cause of the clear cell change. Hemosiderosis was also absent.

### Renal Pathology

A nephrological evaluation of the proband revealed that she had bilateral medullary nephrocalcinosis with small speckled stones in the medullary pyramids and calyceal tips of both kidneys. The proband had a history of hypocalcemia and was treated with calcium and vitamin D supplements. X‐ray studies revealed mild osteopenia. Hypophosphatemic rickets was not apparent, and her serum phosphorus level was normal. There was no evidence of renal Fanconi syndrome. Specifically, she had no glycosuria, proteinuria, nor hyperuricosuria, and no evidence to suggest renal tubular acidosis.

### Evaluation of Additional Family Members

At the age of 11, the proband's brother had similar complaints; he also had bloating and hepatosplenomegaly during infancy but no evidence of a liver mass. MR elastography scans showed increased liver stiffness consistent with stage 2 to 3 liver fibrosis and an abnormal lobulated contour to the liver. SUAC was undetectable in the urine, but interestingly, delta‐aminolevulinic acid (ALA) levels were increased to 25 nmol/ml (Table 1). The proband's brother also had mild hypercalciuria but no generalized aminoaciduria, glycosuria, nor phosphaturia, and no evidence to suggest renal tubular acidosis. A CT scan of the abdomen and pelvis showed a nonobstructing 5 × 4 mm^2^ renal stone in the lower pole of the left kidney. Echogenicity of the kidneys was normal on ultrasonography.

### Whole Exome Sequencing Findings and Confirmatory Sequencing

Whole exome sequencing was conducted on the proband, her mother, and an unaffected brother in a nontraditional trio (Fig. 1). Among 74 variants identified in 51 genes, sequencing revealed a novel homozygous c.424A > G (p.R142G) missense variant in exon 5 of the *FAH* gene in the proband. This variant has not been observed in approximately 6,500 individuals of European and African American ancestry in the NHLBI Exome Sequencing Project, 1000 Genomes Project, or in over 60,000 individuals in ExAC. In silico prediction algorithms classified the variant as deleterious, probably damaging, and disease causing (SIFT, PolyPhen‐2, and MutationTaster2, respectively). Both the mother and unaffected brother were determined to be carriers of this alteration. No other variants were found in genes associated with constitutional genetic disorders with phenotypic overlap that were excluded through clinical biochemical testing.

The variant was confirmed by Sanger sequencing of exon 5 of *FAH* (GeneDX, Gaithersburg, MD) and was considered to be a strong candidate for a pathogenic variant. Germline DNA from the younger male sibling, who had a similar clinical presentation, was also sequenced and found to have the same homozygous p.R142G variant in *FAH*. Targeted CLIA sequencing for the p.R142G variant revealed that both the mother and father were carriers of this variant. A liver needle biopsy from the deceased sibling was obtained from the pathology lab at the treating hospital. The entire sample was de‐paraffinized, and DNA was extracted for Sanger sequencing. The deceased sister was also found to have the homozygous p.R142G variant in *FAH*.

### Evaluation of Liver Tissue for Somatic Conversion

To examine how the variant affects protein stability, we conducted an immunohistological examination of the FAH protein in the proband's explanted liver (Fig. 2C). This revealed normal FAH staining and expression (Fig. 2C). Extracted DNA from two blocks of formalin fixed paraffin‐embedded liver tissue was sequenced for the p.R142G *FAH* variant (Fig. 2G). We did not observe any sequence reversion in the form of low level mosaicism, suggesting that this variant is not prone to spontaneous reversion as is seen in some patients who have IVS12 + 5g > a, p.G337S, p.Q64H, or p.Q279R homozygous variants in *FAH* [Demers et al., 2003]. However, our investigation was limited by the availability of the explanted liver tissue, and consequently it was not possible to entirely rule out somatic reversion elsewhere in the liver.

### In Silico Modeling of the p.R142G Variant in FAH

The p.R142G variant is located in the catalytic pocket of FAH (Supp. Figs. S1 and S2). To ascertain the potential functional impact of this new *FAH* variant on protein structure, we used computational methods to predict non‐neutral variants [Bordner and Abagyan, 2004]. Using the mouse Fah protein crystal structure, which has over 89% amino acid conservation with the human FAH protein, we examined the potential effects of the p.R142G variant on FAH protein stability and on substrate binding within the catalytic site [Timm et al., 1999].

In one crystal structure (Protein Data Bank Entry: 1QCO), p.R142 forms two hydrogen bonds to fumarate and an acetate ion (a crystallization additive) (Supp. Fig. S2) [Timm et al., 1999]. The calculated interaction energy between p.R142 and fumarate in the 1QCO structure is approximately 4.1 kcal/mol, suggesting that the residue forms stabilizing hydrogen bond interactions with favorable interaction geometry and electrostatic energy that appear necessary for substrate binding within the FAH catalytic site.

### In Vivo Functional Testing in a Somatic Mouse Model

**Figure 3:**
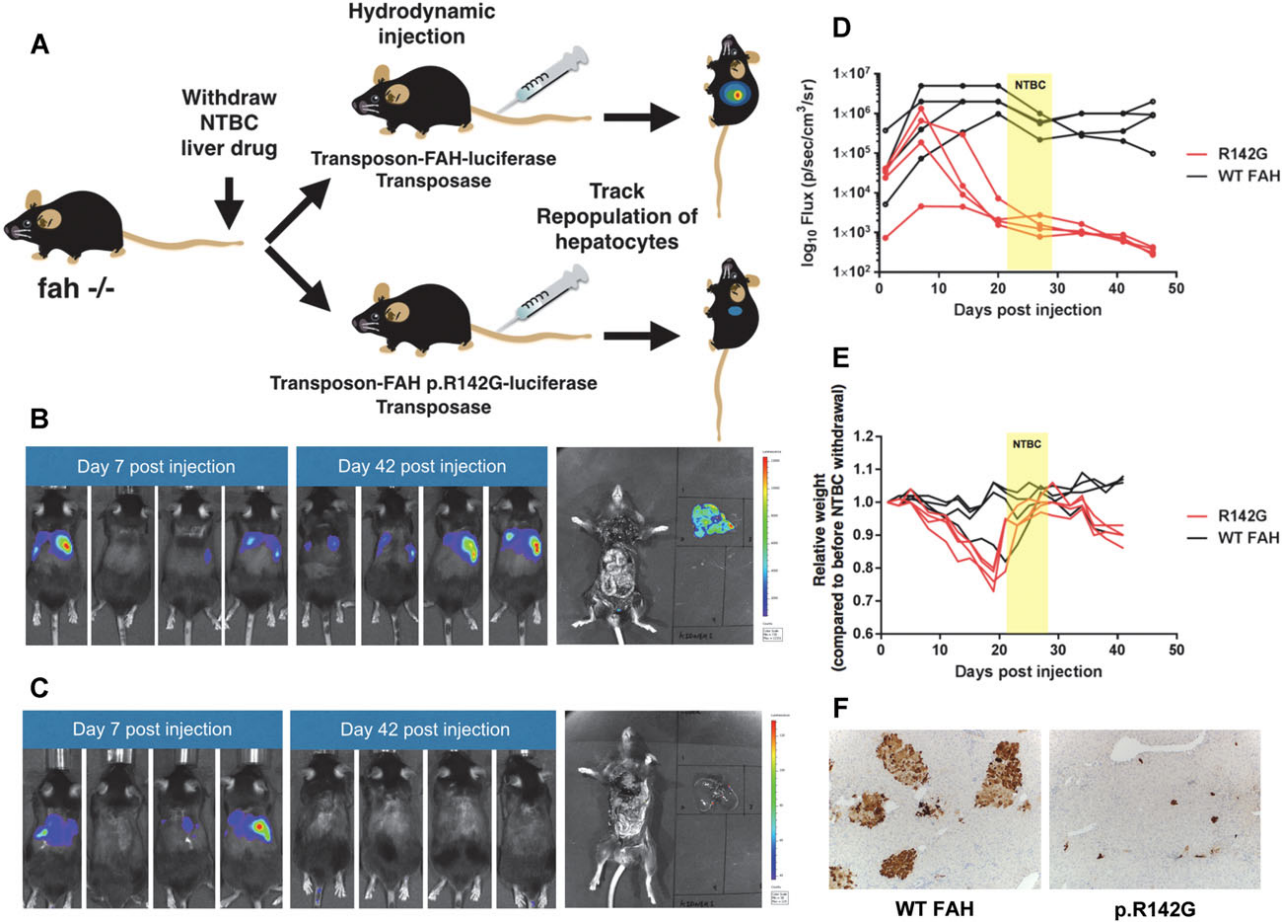
In vivo somatic mouse model shows that the FAH p.R142G variant results in loss of function and fails to rescue the metabolic defect in C57Bl6/6J‐Fah^−/−^ mice. **A**: Fah^−/−^ mice were maintained on NTBC until the day of the hydrodynamic injection procedure. Mice were injected with the bicistronic *Sleeping Beauty* transposon containing either normal human FAH cDNA (pKT2/hFAHIL) or the p.R142G variant FAH cDNA (pKT2/R142G/hFAHIL) as well as luciferase and a separate plasmid providing the *Sleeping Beauty* transposase (pKUb‐SB100X). Three tests were used to infer whether the variant *FAH* cDNA encoded a functional protein that could rescue the hepatotoxicity phenotype found in the FAH‐deficient livers of these mice. **B**: Mice injected with the transposon containing normal human *FAH* cDNA (pKT2/hFAHIL) at 7 and 42 days post‐injection. The liver was removed and imaged alongside the animal at the end of the study. **C**: Mice injected with the transposon containing p.R142G variant *FAH* cDNA (pKT2/R142G/hFAHIL) at 7 and 42 days post‐injection. The liver was removed and imaged alongside the animal at the end of the study. **D**: Changes in total luminescence in mice over time. NTBC was restarted for 1 week (shown in yellow). **E**: Relative weight (compared with before NTBC withdrawal) over time. NTBC was restarted for 1 week (shown in yellow). **F**: FAH immunohistochemistry on liver sections (10×) from mice sacrificed 25 days post‐injection revealed the presence of discrete repopulating hepatocyte foci in mice expressing wild‐type human FAH (left panel). The mice expressing the *FAH* variant had only a few isolated cells expressing FAH across the entire section (right panel).

To establish the pathogenicity of the p.R142G variant in vivo, we used a *Sleeping Beauty* transposon system to express either WT or p.R142G human FAH in conjunction with a luciferase reporter in the livers of C57Bl6/6J‐Fah^−/−^ mice (Fig. 3A). Using this humanized in vivo liver model, our aim was to determine whether and to what degree the mutant enzyme could correct the metabolic deficiency and hepatotoxicity in Fah^−/−^ mice.

For the initial study, equal numbers of mice received hydrodynamic tail vein injections with either pKT2/hFAHIL (*n* = 5) or pKT2/R142G/hFAHIL (*n* = 5) in addition to pKUb‐SB100X, which supplies the transposase. One mouse from each experimental arm was sacrificed at day 25 post‐injection for immunohistochemical studies of the liver. Transient expression of luciferase from the pKT2/hFAHIL and pKT2/R142G/hFAHIL plasmids was evident 24 hr after injection and remained elevated for the first 7 days until luciferase expression steadied to levels reflecting stable integration of the *Sleeping Beauty* transposon vector (Fig. 3D). At 2 weeks after injection, mice expressing the p.R142G variant had rapid onset weight loss (∼20% of initial body weight) (Fig. 3E). Most mice expressing WT FAH lost very little weight and had attained prestudy body weights by this time (Fig. 3E). At 14 days post‐injection both sets of mice were placed back on NTBC (4 mg/l) for 1 week. After NTBC removal, mice expressing the p.R142G variant began to lose weight steadily after about a week. Mice injected with the construct expressing the p.R142G variant had only a transient elevation of luciferase expression that was not maintained after the first 7 days of the study, while mice expressing WT FAH had sustained luciferase expression throughout the course of the study. The animals were followed until the mice expressing the p.R142G variant had lost ∼20% of their total body weight. At this time, both sets of animals were imaged, and a significant difference in luminescence intensity between WT and p.R142G FAH‐expressing animals was noted (Fig. 3B and C, Supp. Fig. S3). These results were confirmed with a second series of mice (Supp. Figs. S4 and S5).

Therapeutic correction of the FAH deficiency provides hepatocytes with a growth and survival advantage. These corrected hepatocytes proliferate extensively and can repopulate large portions of the liver. FAH immunohistochemistry on liver sections revealed multiple discrete repopulating hepatocyte foci in mice expressing WT human FAH 25 days after injection (Fig. 3F). In contrast, mice expressing the p.R142G variant had only a few isolated cells expressing FAH across the entire section. At the end of the study, alanine aminotransferase, alkaline phosphatase, and gamma glutamyl transferase levels were also significantly elevated in mice expressing the p.R142G variant compared with the mice expressing WT FAH (Supp. Fig. S6), which had a reversal of liver damage and normalization of serum liver enzymes.

### In Vitro FAH Enzymatic Assays

**Figure 4:**
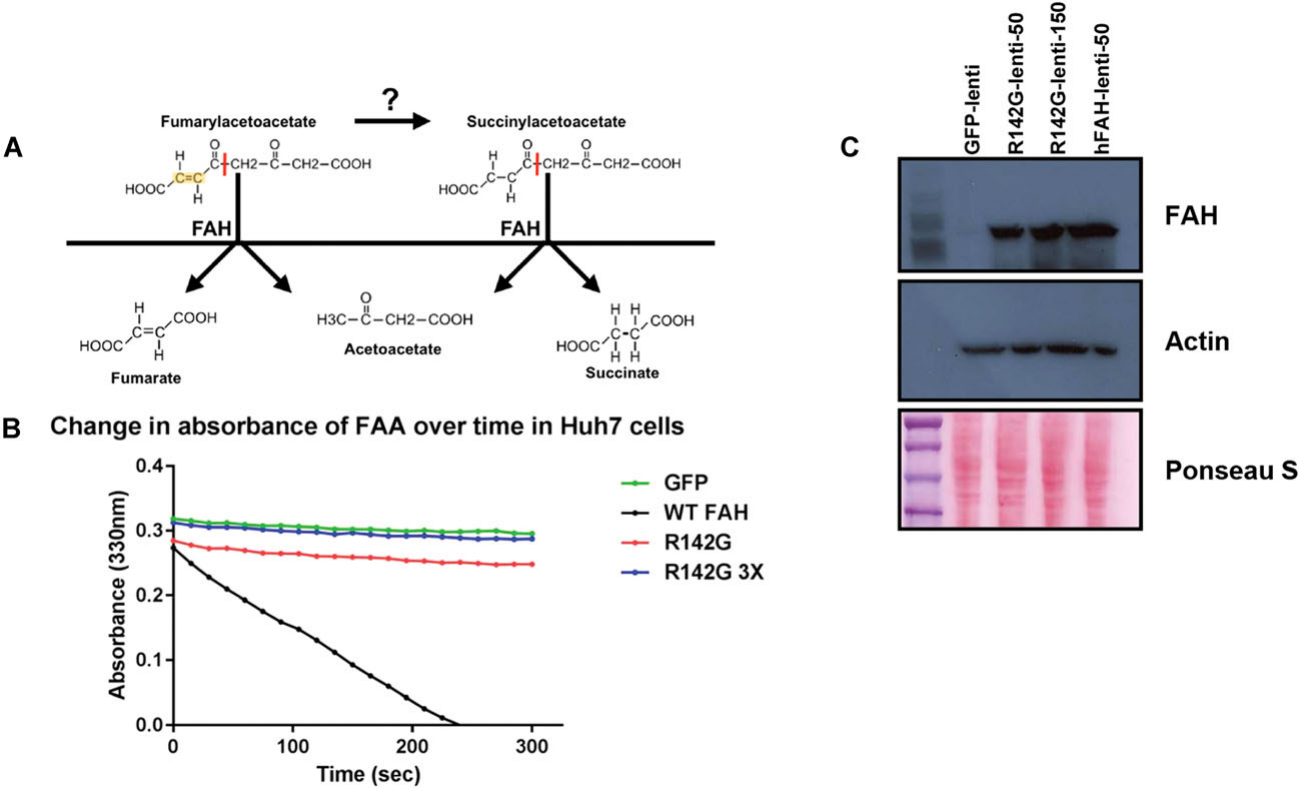
Fumarylacetoacetate hydrolase (FAH) enzyme kinetic assays show that the FAH p.R142G variant is unable to break down fumarylacetoacetate (FAA), the hepto‐ and nephrotoxic substrate in tyrosinemia type 1. **A**: FAA and succinylacetoacetate (SAA) have similar chemical structures; the only difference is the presence of a C–C double bond in FAA (highlighted in yellow). Because of the shape of the catalytic pocket, only the extended transconformations of FAA and SAA can be accommodated. It appears that FAA is bound more tightly by FAH and the rate of dissociation is much slower than for SAA, which may reflect differences in physiological substrate preference. As FAA is the reactive substrate that causes liver damage, the ability of FAH to process FAA (and not SAA) is thus a clinically relevant molecular assay for predicting hepatotoxicity potential for sequence variants in this enzyme. **B**: Enzyme kinetic assay for FAH activity measures the decrease in absorbance of FAA (330 nm) over time. Lentiviral constructs (green fluorescent protein control, human FAH, and p.R142G variant FAH) were used to transduce the Huh7 hepatocellular carcinoma cell line. Huh7 cells transduced with the wild‐type human FAH construct were able to break down FAA as evidenced by the decrease in absorbance at 330 nm over time. Huh7 cells transduced with the p.R142G variant lentivirus had a decrease in absorbance that was identical to that of the green fluorescent protein control, indicating that the variant was a functional null allele and is therefore incapable of breaking down FAA. Huh7 cells transduced with three times the amount of p.R142G‐lentivirus also did not show a measurable decrease in absorbance at 330 nm compared with the green fluorescent protein‐lentivirus control. **C**: Western blot analysis of FAH (∼46 kDa) showing that the two lentivirus constructs expressed equivalent levels of protein in Huh7 cells transduced at the same multiplicity of infection. Lane 1 is the green fluorescent protein control. Lanes 2 and 3 are the R142G variant FAH lentivirus. Lane 4 is the wild‐type human FAH lentivirus.

Huh7 cells were transduced with lentivirus expressing WT, the p.R142G variant of FAH, or a green fluorescent protein (GFP) control at the same calculated multiplicity of infection. Western blot confirmed that the Huh7 cell line has no endogenous FAH expression (data not shown). Cells were expanded and harvested for enzyme kinetic analysis. By measuring the decrease in absorbance of FAA at 330 nm, we were able to determine that the p.R142G variant FAH protein was incapable of breaking down FAA and had a decrease in absorbance in both cell lines that was identical to the GFP‐lentivirus negative control (Figs. 4A and B). Huh7 cells transduced with three times the amount of p.R142G‐lentivirus did not show a measurable decrease in absorbance at 330 nm compared with the GFP‐lentivirus control. Western blot analysis of FAH showed that the two lentivirus constructs expressed similar levels of protein in cells transduced at the same multiplicity of infection (Fig. 4C). These studies confirm that the p.R142G variant FAH protein is unable to breakdown FAA, the hepatotoxic substrate in TYRSN1.

### ALA is Elevated in Some TYRSN1 Patients on NTBC who have Undetectable SUAC

**Figure 5:**
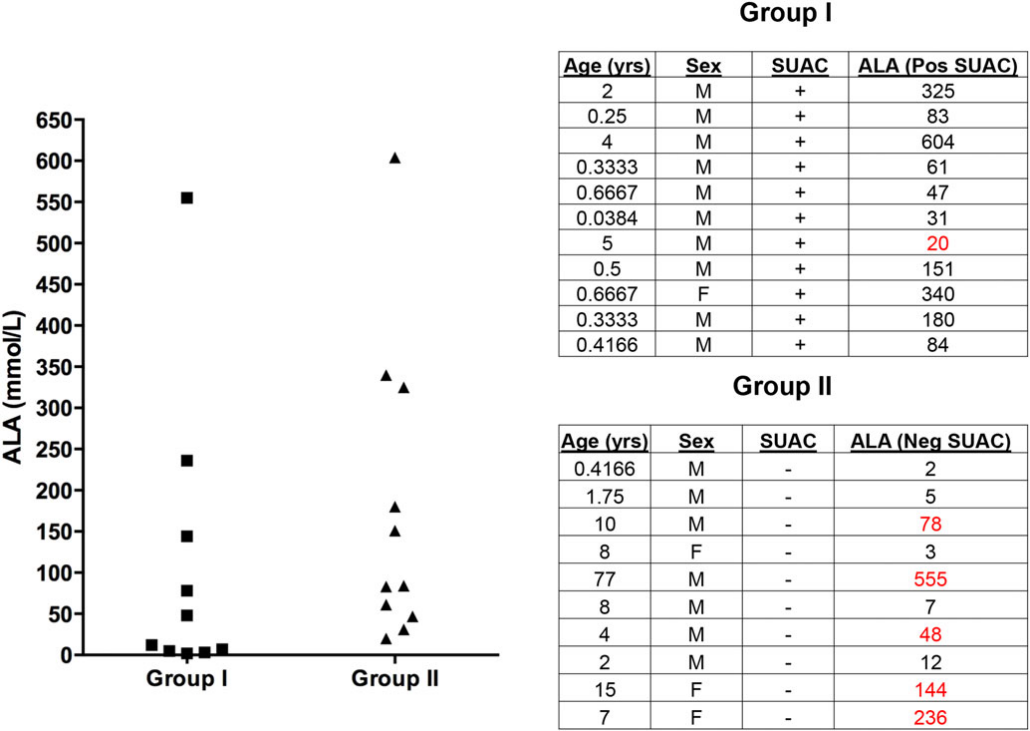
Delta‐aminolevulinic acid (ALA) is elevated in the absence of detectable succinylacetone (SUAC) in some TYRSN1 patients. ALA levels (nmol/mL) in TYRSN1 patients with undetectable SUAC (Group I) and patients with elevated SUAC (Group II) from a Mayo Clinic cohort. A number of patients being treated with NTBC from group I had undetectable SUAC but were found to have elevated ALA (highlighted in bold) (reference ranges: <1 year: ≤10 nmol/ml, 1–17 years: ≤20 nmol/ml). One patient in group II (indicated with an asterisk) was found to have normal ALA levels despite having detectable SUAC.

SUAC is a potent inhibitor of delta‐ALA, and it is intriguing that we observed slightly elevated levels in the proband's affected brother. Interestingly, we have observed patients with TYRSN1 who are on NTBC and who have undetectable levels of SUAC can still have elevated ALA levels, which is a previously unreported finding that supports the idea of unexpected biochemical complexity in diverse patients with TYRSN1 (Tortorelli S, unpublished data) (Fig. 5). While the explanation for slightly elevated ALA in our patient is unknown, lead poisoning could also lead to this finding and unfortunately testing was not performed to rule this out as a cause.

## Discussion

TYRSN1 is an inborn error of tyrosine metabolism that can lead to life‐threatening consequences if not recognized and treated early in life. Therefore, efficient newborn and carrier screening protocols are essential to ensure early detection and effective treatment of the disease [Angileri et al., 2015]. Tyrosine is not an optimal screening marker for TYRSN1 because it is not sensitive enough to detect all cases, so a more targeted approach that measures the level of SUAC in the original blood spot used for newborn screening has been proposed [American College of Medical Genetics Newborn Screening Expert Group, 2006; Turgeon et al., 2008]. However, a substantial number of cases likely remain undiagnosed partly because of inadequate screening for TYRSN1 [Angileri et al., 2015].

Two prior cases have been described in which children who had TYRSN1 had undetectable levels of SUAC. A child described by Cassiman et al. (2009) was found to have a novel c.103G > A (p.A35T) variant in *FAH* and had elevations in the levels of tyrosine (651 nmol/ml), methionine (1,032 nmol/ml), and alpha‐fetoprotein (29,723 ng/ml), which were diagnostic in the absence of elevated SUAC. In another case, a 15‐month‐old patient who had the French Canadian common variant in *FAH* (IVS12 + 5G‐A) and another previously unreported *FAH* variant (not noted) was found to have undetectable SUAC levels [Rinaldo et al., 2006]. These examples suggest that not all TYRSN1 patients have altered biochemical markers, such as elevated SUAC.

Somatic reversion within the liver is one potential mechanism that could explain the liver damage along with the atypical biochemical presentation observed in this family. While it was not possible for us to rule this out completely in our studies, evidence from transplanted TYRSN1 cases suggests that this is unlikely. TYRSN1 patients who have undergone orthotopic liver transplantation continue to exhibit elevated urine and plasma SUAC levels for the duration of post‐transplant follow‐up associated with low to normal ALA dehydratase activity [Bartlett et al., 2013]. This is likely due to the continued production of SUAC by the renal proximal tubules.

In silico modeling suggests that the p.R142 residue plays an important role in the binding of both FAA and SAA within the catalytic pocket of the enzyme. Given that the p.R142G variant is unable to break down FAA and biochemical testing for SUAC and other metabolites was negative, we hypothesize that the variant form may still bind and catabolize SAA, resulting in undetectable levels of SUAC. However, it is unable to break down FAA, the hepatotoxic substrate in TYRSN1, which leads to liver damage and the development of HCC. Some of the FAA that accumulates is reduced to SAA, which could be catabolized by the p.R142G variant to form succinate and acetoacetate without the accumulation of upstream metabolites. Additional studies will be required to determine whether the variant form is able to breakdown SAA.

Because of the overlap in disease phenotypes and the variability in clinical presentations, WES is being increasingly utilized for both faster and more cost‐efficient diagnosis. Through WES we were able to identify a novel variant in *FAH* in a family with cirrhosis and a predisposition to HCC. We used an in vivo somatic model and transposon delivery system to assay the pathogenicity of this suspected causal variant in a clinically relevant time frame (months). Due to the confounding clinical presentation seen in members of this family, functional validation was essential for unraveling the role of this novel variant. It remains to be determined whether the variant identified in this family is more widespread, particularly within geographically related populations where there is a high rate of endogamy [Imtiaz et al., 2011]. We recently identified a patient who had compound heterozygous variants in FAH, including the p.R142G variant that was identified in a family with Lebanese and Italian ancestry (Scott CR, personal communication). It is therefore interesting to speculate that there may be other individuals harboring this variant that evaded detection efforts due to current newborn screening practices. Molecular testing of patients who have clinical presentations suggestive of TYRSN1, including pediatric cryptogenic cirrhosis, is thus indicated for improving the overall comprehensive clinical diagnosis of patients with TYRSN1.

## Author Contributions

P.R.B., R.D.H., E.W.K., L.R.R., and S.C.E. designed the study. P.R.B., R.D.H., R.A.N., N.H.G., D.L.K., A.J.B., R.C., J.B.M., M.R., R.P.G., M.S.T., S.T., C.R.S., N.M.L., D.S.M., D.O., W.A., M.G., D.K.G., M. E., K.J.C., P.S.A., E.W.K., L.R.R., and S.C.E. gathered the data. P.R.B., R.D.H., E.W.K., and S.C.E. analyzed the data. P.R.B., E.W.K, and S.C.E. wrote the paper.

## Supporting information

Disclaimer: Supplementary materials have been peer‐reviewed but not copyedited.

Supplementary Methods and Figures
