# Supplementary material for: Silent Tyrosinemia Type I Without Elevated Tyrosine or Succinylacetone Associated with Liver Cirrhosis and Hepatocellular Carcinoma
Source: Hum Mutat. 2016 Aug 8;37(10):1097–105. doi: 10.1002/humu.23047 (PMC5108417; doi:10.1002/humu.23047)
Supplement: Supplementary file 1 — Supplementary Methods and Figures [file HUMU-37-1097-s001.pdf]

Supplementary Methods and Figures

Silent tyrosinemia type I without elevated tyrosine or succinylacetone associated with liver cirrhosis and hepatocellular carcinoma

Patrick R. Blackburn[1], Raymond D. Hickey[1], Rebecca A. Nace[1], Nasra H. Giama[1], Daniel L. Kraft[1], Andrew J. Bordner[2], Roongruedee Chaiteerakij[1,3], Jennifer B. McCormick[1], Maja Radulovic[1], Rondell P. Graham[1], Michael S. Torbenson[1], Silvia Tortorelli[1], C. Ronald Scott[4], Noralane M. Lindor[5], Dawn S. Milliner[1], Devin Oglesbee[1], Wafa'a Al-Qabandi[6], Markus Grompe[7], Dimitar K. Gavrilov[1], Mounif El-Youssef[1], Karl J. Clark[1], Paldeep S. Atwal[8], Lewis R. Roberts[1], Eric W. Klee[1]* and Stephen C. Ekker[1]*

*Corresponding authors. E-mail: ekker.stephen@mayo.edu and klee.eric@mayo.edu

Supplementary Data:

Additional Clinical Testing

During the clinical workup at the treating institution, glycogen storage disorder type III and other glycogen storage disorders were excluded through biochemical testing of enzymatic activity in the explanted liver. Glycogen content and structure, debranching enzyme, branching enzyme, glucose-6-phosphatase, and phosphorylase enzymes all were within normal ranges. Mannose phosphate isomerase gene sequencing was conducted to test for congenital disorder of glycosylation 1B, but no variants were detected. Other constitutional genetic syndromes including galactosemia, fructosemia, Alagille syndrome, Wilson disease, Wolman disease,

Gaucher's disease, cerebrotendinous xanthomatosis, polyol disorders, and Fanconi syndrome were excluded through further testing.

Mouse experiments

A second series of Fah-/- mice received hydrodynamic tail vein injections with either pKT2/hFAHIL or pKT2/R142G/hFAHIL in addition to pKUb-SB100X as in the previous experiment. When mice surpassed the > 20% weight loss threshold (Mayo Clinic Institutional Animal Care and Use Committee mandated) they were euthanized rather than being placed back on NTBC. The weights of the mice followed a similar trend to the mice in the first experiment. Mice expressing the wild-type fumarylacetoacetate hydrolase (WT FAH) had a slight decrease in initial weight, but they recovered or exceeded their day 0 weights by the end of the study (day 35), indicating therapeutic correction of FAH deficiency. Mice expressing mutant FAH lost weight more slowly, but all had lost >20% total weight by day 30 with no evidence of recovery (**Fig. S4**). By the end of the study a significant difference in luminescence intensity between WT and mutant FAH expressing animals was noted (**Fig. S5**).

FAH immunohistochemistry

Two mice with comparable luciferase expression, one expressing WT FAH and another expressing the mutant FAH, were sacrificed at 13 days post-injection to compare FAH expression early in the repopulation process. Even early on, the repopulation process appeared to proceed much more rapidly in the mouse expressing WT FAH. Large areas of repopulating hepatocytes were observed, whereas hepatocytes in the mouse expressing mutant FAH exhibited a much more diffuse and scattered pattern of FAH expression.

Whole exome sequencing, analysis, and variant discovery

DNA extracted from blood derived from the proband, the proband's mother, and an unaffected sibling was enriched for exonic DNA using Sure Select XT All Exon 50 MB Capture from Agilent followed by deep sequencing on the Illumina HiSeq 2000 sequencing system. Exon capture and deep sequencing was completed at the Mayo Clinic Medical Genome Facility. A non-traditional trio analysis was performed because of difficulties obtaining a sample from the father of the proband.

Sequence alignment and variant calling was done using the Mayo Clinic DNA Sequencing Analytic Pipeline, Genome GPS. Briefly, 100-bp paired end reads were aligned to hg19 using Novoalign (Novocraft Technologies, Malaysia), and the quality of sequencing chemistry was evaluated using FastQC (http://www.bioinformatics.babraham.ac.uk/projects/fastqc/). Realignment and recalibration steps used Best Practice Variant Detection v3 recommendations implemented in the Genome Analysis Toolkit, and all germline variant calling (both single nucleotide and small insertions and deletions) were called through the Genome Analysis Toolkit (McKenna, et al., 2010). After alignment, PCR duplication rates and percent reads mapped on target were used to assess the quality of the sample preps.

Causal variants in this study were identified using Qiagen's Ingenuity® Variant Analysis™ software (www.ingenuity.com/variants) (QIAGEN, Redwood City, CA). Raw single sample Variant Call Format files were uploaded onto Ingenuity's exome project server.

Starting with 451,936 variants spanning 19,679 genes, the whole exome sequence data was filtered to contain only variants with high-quality calls (at least 20.0) in both of the cases and controls. Variants were excluded if they had a minor allele frequency greater than or equal to 3.0% in the 1000 Genomes Project, the NHLBI ESP exomes, or ExAC. Variants predicted to be pathogenic or likely pathogenic (resulting from frameshift, in-frame indel, missense, or stop codon changes) were kept. Analysis was restricted to unique hemizygous, homozygous, haploinsufficient, and compound heterozygous mutations that occurred in the proband but were not found in the mother or unaffected brother and were consistent with a predicted autosomal recessive mode of inheritance. Biological filtering was also applied and genes known or predicted to affect the development of hepatocellular carcinoma were kept.

Variants were visualized using the Integrated Genomics Viewer (Broad Institute, Boston, MA).

Genomic PCR and confirmation by Sanger sequencing

Genomic DNA isolated from whole blood was amplified using the following primer pair: 5'-GGCTGAGCTCTGGATGTGTAA-3' (sense, intron 4) and 5'-CCTTCGATCCCACAGCAAGT-3' (anti-sense, intron 5). The PCR reaction contained, in a total volume of 50 µl, 1 µl of diluted extracted DNA, 5 µl of 10X High Fidelity PCR buffer, 1 µl of 10 mM deoxynucleotide mixture, 0.2 µM of each primer, 2 µl of 50 mM $MgCl_2$ and 0.2 µl Platinum® Taq High Fidelity DNA Polymerase (Life Technologies, Carlsbad, CA). Thermocycler conditions included an initial denaturation at 94ºC for 3 minutes, followed by 35 cycles of 94°C for 30 seconds, 58°C for 30 seconds, 68°C for 30 seconds, and a 5 min final extension at 72°C. The resulting 415-bp amplicon was prepared for direct sequencing with the

ABI PRISM™ Big Dye Terminator Cycle Sequencing Ready Reaction Kit using AmpliTaq® DNA Polymerase and sequenced on the Applied Biosystems 3730xl DNA Analyzer (Applied Biosystems, Foster City, CA). Similarly, DNA was extracted from formalin fixed paraffin embedded tissue sections from the explanted liver using the QIAamp DNA Mini Kit (QIAGEN, Redwood City, CA) and amplified as described above.

For the deceased sister, genomic DNA isolated from a formalin fixed paraffin-embedded liver needle biopsy was amplified using the following primer pair: 5'-ACACTCGTGGCTCCAGTGCTT -3' (sense, intron 4) and 5'-AGCTCCTGAGACAAATATGCTG-3' (anti-sense, intron 5). The PCR reaction contained, in a total volume of 10 µl, 3 µl of diluted extracted DNA, 2 µl of 5X KAPA2G buffer A(with $MgCl_2$), 0.2 µl of 10 mM deoxynucleotide mixture, 1.5 µl of 1.25 µM of each primer, and 0.08 µl KAPA2G Robust Hotstart DNA polymerase (KAPA Biosystems). Thermocycler conditions included an initial denaturation at 98ºC for 3 minutes, followed by 15 cycles of 95°C for 30 seconds, 64.5°C for 30 seconds, and 72°C for 1 min, followed by 20 cycles of 95°C for 30 seconds, 58°C for 30 seconds, and 72°C for 1 min, followed by 10 min final extension at 72°C. The resulting amplicon was a 297-bp product and was prepared for direct sequencing with the ABI PRISM™ Big Dye Terminator Cycle Sequencing Ready Reaction Kit using AmpliTaq® DNA Polymerase on the Applied Biosystems 3730xl DNA Analyzer.

Patient FAH immunohistochemistry

Livers harvested from mice at the completion of the study were fixed in 10% neutral buffered formalin over night at 4°C. The next day formalin was replaced with 70% ethanol, and the liver

sections were kept at room temperature until they were embedded in paraffin. 5 μm sections were cut and mounted serially onto Superfrost slides (Fisher Scientific, Pittsburgh, PA). One representative slide from one or more liver lobes was stained with hematoxylin and eosin. In addition several slides were stained for FAH protein by immunohistochemistry. The mouse immunohistochemistry staining procedure was performed using the Leica Bond III stainer (Leica, Buffalo, IL). The tissue slides were dewaxed and retrieved on-line using the following reagents: Bond Dewax (Leica, Buffalo, IL) and Epitope Retrieval 2 (Leica, Buffalo, IL). Tissue slides were retrieved for 20 minutes and blocked using Rodent Block M (Biocare RBM961) for 30 minutes. The primary rabbit polyclonal anti-rat FAH antibody (designated FAH Grompe OR026 rabbit polyclonal, Markus Grompe, OHSU) was used at a 1:2000 dilution and incubated for 15 minutes (Wang, et al., 2002). The detection system used, Polymer Refine Detection System (Leica, Buffalo, IL), includes a hydrogen peroxidase block, secondary antibody polymer, diaminobenzidine and hematoxylin. Once stained, slides were rinsed for 5 minutes in tap water. Slides were also dehydrated in increasing concentrations of ethyl alcohol and xylene prior to immersion in xylene-based media and sandwiched by a coverslip.

Molecular modeling of the FAH enzyme

Molecular mechanics simulations were used to predict the geometry and relative stability of point mutants to detect nonneutral mutations (Bordner and Abagyan, 2004). All calculations were performed using biased probability Monte Carlo sampling with the ICM program (Molsoft LLC) (Abagyan and Totrov, 1994). We first generated a structural model with idealized covalent geometry starting from the high-resolution X-ray structure of mouse Fah in complex with the natural catalytic products, acetoacetate and fumarate (Protein Data Bank entry 1QCO) (Timm, et

al., 1999). The change in binding energy was then estimated as a weighted sum of energy components calculated after performing local structure optimization following the procedure described in Bordner et al (Bordner and Abagyan, 2004).

FAH is a carbon-carbon bond hydrolase composed of two subunits that form a homodimer (Timm, et al., 1999). FAH shows a high degree of amino acid conservation across species (~47% overall), with complete conservation of the residues forming the catalytic pocket (Timm, et al., 1999). The catalytic site is located near the dimer interface and is formed by a unique glutamic acid-histidine-water catalytic triad (**Fig. S1**) (Timm, et al., 1999). Using the mouse Fah protein crystal structure, which has over 89% amino acid conservation with the human FAH protein, we examined the potential effects of the c.424A>G mutation on FAH protein stability and on substrate binding within the catalytic site (Timm, et al., 1999).

R142 does not directly interact with the other subunit at the dimer interface and therefore was not predicted to have a significant effect on quaternary structure. The conservation and predicted impact of this missense mutation was further investigated using MutationTaster (http://www.mutationtaster.org/) (Schwarz, et al., 2014). The R142 residue is highly conserved across species. The p.R142G missense mutation was predicted to be disease causing by the Bayes classifier employed by MutationTaster (Schwarz, et al., 2014).

Due to the shape of the catalytic pocket, only the extended *trans* conformations of fumarylacetoacetate and succinylacetoacetate can be accommodated (Timm, et al., 1999). The *cis* isomer of fumarylacetoacetate, maleylacetoacetate is sterically hindered due to Y128, Y244,

and R142 at the entrance to the catalytic pocket (**Fig. S1**) (Timm, et al., 1999). These three residues form a hydrogen bond network that restricts active site entry (Timm, et al., 1999). Active site binding of fumarylacetoacetate within the active site has been shown to alter the conformation of Y128, Y244, and R142, which may accompany changes to the catalytic site that allow formation and breakdown of the proposed tetrahedral alkoxy intermediate (**Fig. S1**) (Timm, et al., 1999).

Bicistronic *Sleeping Beauty* vector construction

pKT2/FAHIL is a bicistronic *Sleeping Beauty* transposon vector that contains a chicken beta-actin promoter that drives expression of mouse Fah and firefly luciferase, which are separated by the Encephalomyocarditis virus internal ribosome entry site (Wangensteen, et al., 2008). The pKT2/FAHIL vector was digested with EcoRI and NotI, removing the mouse FAH cDNA. The human FAH cDNA clone (I.M.A.G.E. ID# 3139988, BC002527, Source BioScience, Nottingham, UK) was amplified using primers that contained compatible EcoRI and NotI digestion sites using Platinum® Taq High Fidelity DNA Polymerase (Life Technologies, Carlsbad, CA). The amplified product was cloned into the TOPO TA vector (Life Technologies, Carlsbad, CA). To create the mutant cDNA, site directed mutagenesis was performed using the following overlapping oligos:

5’-
GCATGCTACCAACGTCGGAATCATGTTC**G**GGGACAAGGAGAATGCGTTGATGCCAA
A-3’

5’-

TTTGGCATCAACGCATTCTCCTTGTCCC**C**GAACATGATTCCGACGTTGGTAGCATGC-

3’

Both the human FAH cDNA and the p.R142G mutant version were inserted into the pKT2/FAHIL construct using T4 DNA ligase (NEB, Ipswich, MA) to create pKT2/hFAHIL and pKT2/R142G/hFAHIL, respectively. Transposase was supplied by the pKUb-SB100X, which has been described previously and utilizes the ubiquitin C promoter (Carlson, et al., 2011).

Mouse blood collection

Blood was collected from the mice by submandibular puncture using an E-Zject 21-gauge lancet (Medi-Point, Mineola, NY). Approximately 0.2 mL of blood was collected in lithium heparin microtainer tubes (BD Medical, Franklin Lakes, NJ) for a baseline measurement of liver function 1 week prior to hydrodynamic injections. Mice were bled every two weeks thereafter and immediately prior to tissue harvest. Blood was kept covered on ice until it could be processed. The blood samples were spun down at ~14,000 *g* for 1 minute, and the plasma was transferred to a clean 1.5 mL microcentrifuge tube. 100 µL of plasma was immediately run on the VetScan Chemistry Analyzer (Abaxis, Inc. Union City, CA). The remaining plasma was frozen at -80˚C.

Mouse liver panel

The VetScan Mammalian Liver Profile (Abaxis, Inc. Union City, CA) was used to analyze plasma collected from mice for *in vitro* quantitative determinations of alanine aminotransferase, alkaline phosphatase, total bilirubin, and gamma glutamyl transferase. Baseline measurements

were collected one week prior to hydrodynamic injections and every two weeks following the injections to monitor metabolic correction of FAH deficiency. Sample collection and preparation was conducted in accordance with the manufacturer's instructions.

Lentiviral vector production

pSIN-CSGWdlNotl-based lentiviral vectors were produced from the parent vector pSIN-CSGWdlNotl-SFFV-EGFP that contained the EGFP transgene under the control of the spleen focus forming virus (SFFV) promoter (Nelson, et al., 2009). *FAH* or mutant *FAH* (p.R142G) was cloned into this vector in place of EGFP to produce the LV-SFFV-FAH expression constructs. Briefly, both the *FAH* or the mutant gene were amplified using primers (FAH_BamHI_F 5'-AAAGGATCCAGCATGTCCTTCATCCCGGTG-3' and FAH_NotI_R 5'-TTTGCGGCCGCTCATGATGGCAGGAGAGCAGGCA-3') that contained compatible BamHI and NotI (NEB, Ipswich, MA) digestion sites using Platinum® Taq High Fidelity DNA Polymerase (Life Technologies, Carlsbad, CA). The amplified product was cloned into the TOPO TA vector (Life Technologies, Carlsbad, CA). Plasmid DNA was isolated using the QIAprep Spin Miniprep Kit (QIAGEN, Redwood City, CA). Plasmids were digested with BamHI and NotI at 37°C and gel isolated using the QIAEX II Gel Extraction Kit (QIAGEN, Redwood City, CA). pSIN-CSGWdlNotl-SFFV-EGFP was digested with BamHI and NotI, and the receiver plasmid was gel isolated as above. Fragments were ligated using T4 DNA ligase (NEB, Ipswich, MA). To generate viral vectors, the FAH expression constructs, along with the packaging plasmid pCMVR8.91 and the vesicular stomatitis virus glycoprotein G (VSV-G)-expressing plasmid, pMD.G, were transiently transfected into 293 cells using polyethylenimine (Polysciences Inc, Warrington, PA) (Zufferey, et al., 1997). Transfected cells were washed after

16 hours with media, and grown for another 48 hours. The supernatants were then harvested and passed through a 0.45 μm filter. Vector supernatants were concentrated by ultracentrifugation (25,000RPM, 1.5 hours at 4°C) and resuspended in serum-free media (OptiMEM, Life Technologies, Carlsbad, CA), aliquoted, and stored at -80°C. Viral titers were calculated using the p24 lentivirus titration kit (Clontech Laboratories, Inc., Mountain View, CA). Titers for GFP, hFAH, and R142G were calculated to 3.0 E-10, 3.3 E-10, and 2.0 E-10 lentiviral particles/ml.

Supplementary References

Abagyan R, Totrov M. 1994. Biased probability Monte Carlo conformational searches and electrostatic calculations for peptides and proteins. Journal of molecular biology 235(3):983-1002.

Bordner AJ, Abagyan RA. 2004. Large-scale prediction of protein geometry and stability changes for arbitrary single point mutations. Proteins 57(2):400-13.

Carlson DF, Garbe JR, Tan W, Martin MJ, Dobrinsky JR, Hackett PB, Clark KJ, Fahrenkrug SC. 2011. Strategies for selection marker-free swine transgenesis using the Sleeping Beauty transposon system. Transgenic research 20(5):1125-37.

McKenna A, Hanna M, Banks E, Sivachenko A, Cibulskis K, Kernytsky A, Garimella K, Altshuler D, Gabriel S, Daly M, DePristo MA. 2010. The Genome Analysis Toolkit: a MapReduce framework for analyzing next-generation DNA sequencing data. Genome research 20(9):1297-303.

Nelson TJ, Martinez-Fernandez A, Yamada S, Mael AA, Terzic A, Ikeda Y. 2009. Induced pluripotent reprogramming from promiscuous human stemness related factors. Clinical and translational science 2(2):118-26.

Schwarz JM, Cooper DN, Schuelke M, Seelow D. 2014. MutationTaster2: mutation prediction for the deep-sequencing age. Nature methods 11(4):361-2.

Timm DE, Mueller HA, Bhanumoorthy P, Harp JM, Bunick GJ. 1999. Crystal structure and mechanism of a carbon-carbon bond hydrolase. Structure 7(9):1023-33.

Wang X, Montini E, Al-Dhalimy M, Lagasse E, Finegold M, Grompe M. 2002. Kinetics of liver repopulation after bone marrow transplantation. The American journal of pathology 161(2):565-74.

Wangensteen KJ, Wilber A, Keng VW, He Z, Matise I, Wangensteen L, Carson CM, Chen Y, Steer CJ, McIvor RS, Largaespada DA, Wang Xet al. 2008. A facile method for somatic, lifelong manipulation of multiple genes in the mouse liver. Hepatology 47(5):1714-24.

Zufferey R, Nagy D, Mandel RJ, Naldini L, Trono D. 1997. Multiply attenuated lentiviral vector achieves efficient gene delivery in vivo. Nature biotechnology 15(9):871-5.

Supplementary Figures:

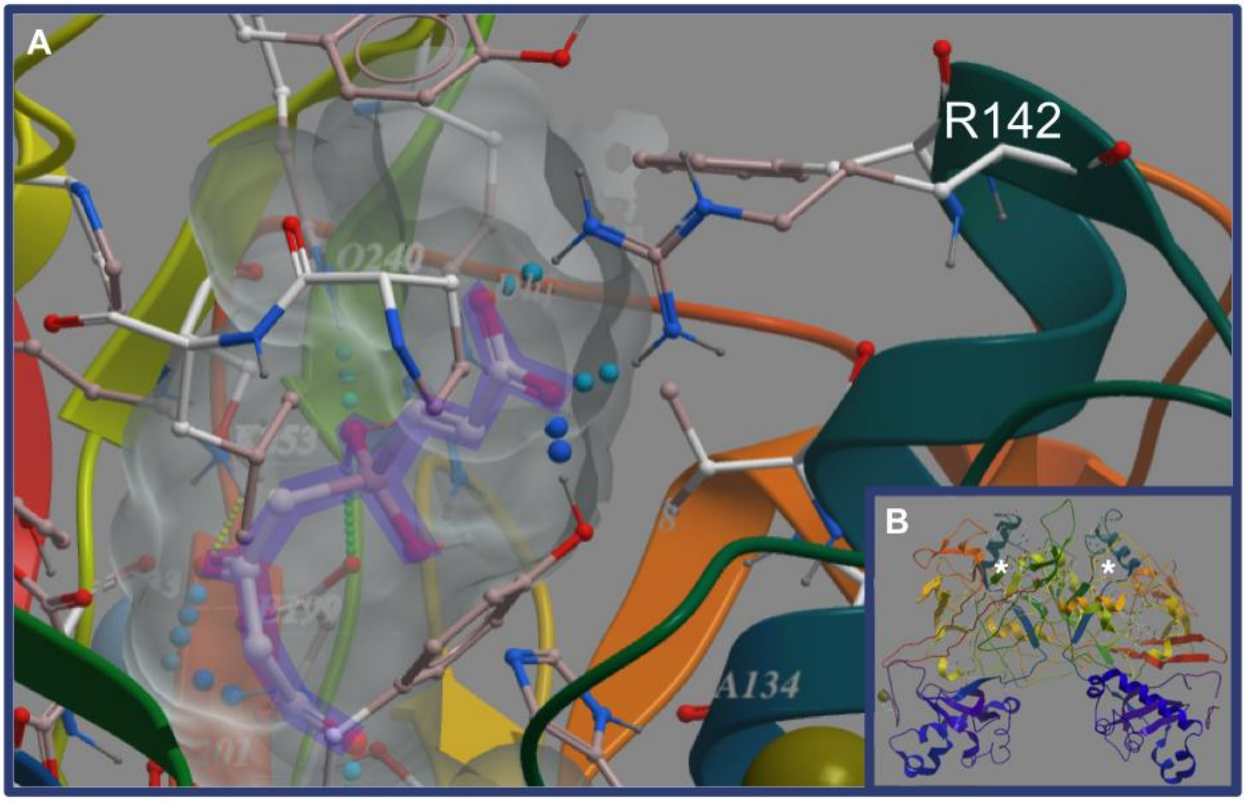

Supp. Figure S1. *In silico* structural modeling suggests that the p.R142G variant is expected to significantly disrupt fumarylacetoacetate hydrolase substrate binding.

(A) Structural modeling using the published crystal structure (Fah, Protein Data Bank Entry: 2HZY) shows R142 contacts the succinylacetoacetate transition state mimic CEHPOBA (4-(2-carboxyethyl)-hydroxyphosphinyl-3-oxobutyrate) (**purple**) at the entrance to the catalytic pocket. The calculated interaction energy between R142 and the transition state mimic indicates a significant stabilizing interaction. Also, because these interactions with R142 are highly conserved, the p.R142G variant was expected to significantly disrupt substrate binding.

(B) Quaternary structure of Fah (Protein Data Bank Entry 2HZY). Fah is a homodimer with two catalytic sites (indicated by *).

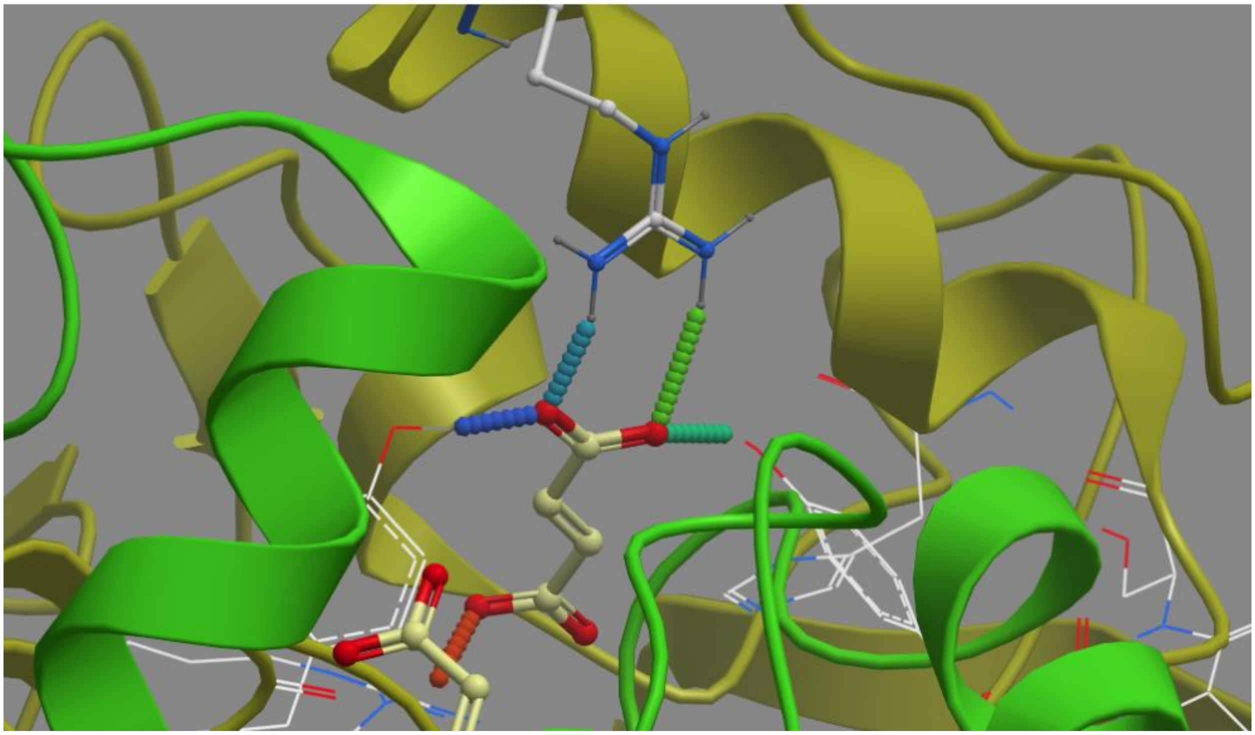

Supp. Figure S2. Calculated interaction energy between FAH p.R142 and fumarate.

In one potential catalytic mechanism, the fumarylacetoacetate substrate is initially bound by the protonated side chain of R142 in complex with a Ca^{2+} ion within the catalytic pocket of the enzyme.[5] One crystal structure (Protein Data Bank Entry: 1QCO) has bound fumarate and acetoacetate products. The p.R142G missense variant in this protein results in the replacement of a hydrophilic residue (arginine) with a neutral one (glycine). In 1QCO, R142 forms two hydrogen bonds to fumarate. The calculated interaction energy between R142 and fumarate in the 1QCO structure is approximately 4.1 kcal/mol, suggesting stabilizing hydrogen bond interactions with favorable interaction geometry and electrostatic energy. Because these substrate interactions with R142 are highly conserved, the R142G mutation is expected to significantly disrupt binding. R142 does not directly interact with the other subunit at the dimer interface and therefore was not predicted to have a significant effect on quaternary structure.

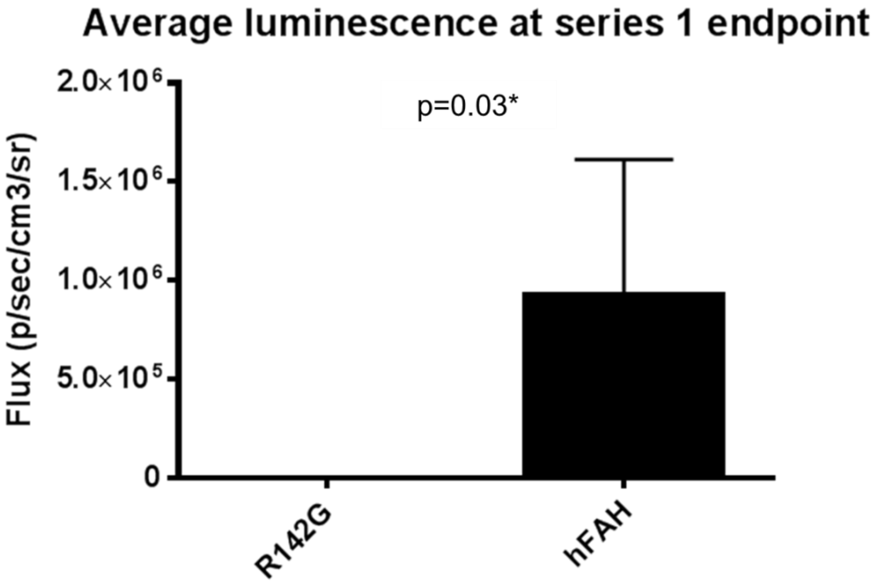

Supp. Figure S3. Average luminescence at the time of tissue harvest for the first series of mice.

Luminescence intensity in mice expressing wild type or p.R142G mutant fumarylacetoacetate hydrolase at the study endpoint (tissue harvest). A significant difference was detected between these two groups, indicating correction of the fumarylacetoacetate hydrolase deficiency in mice expressing the wild-type form but not the mutant form.

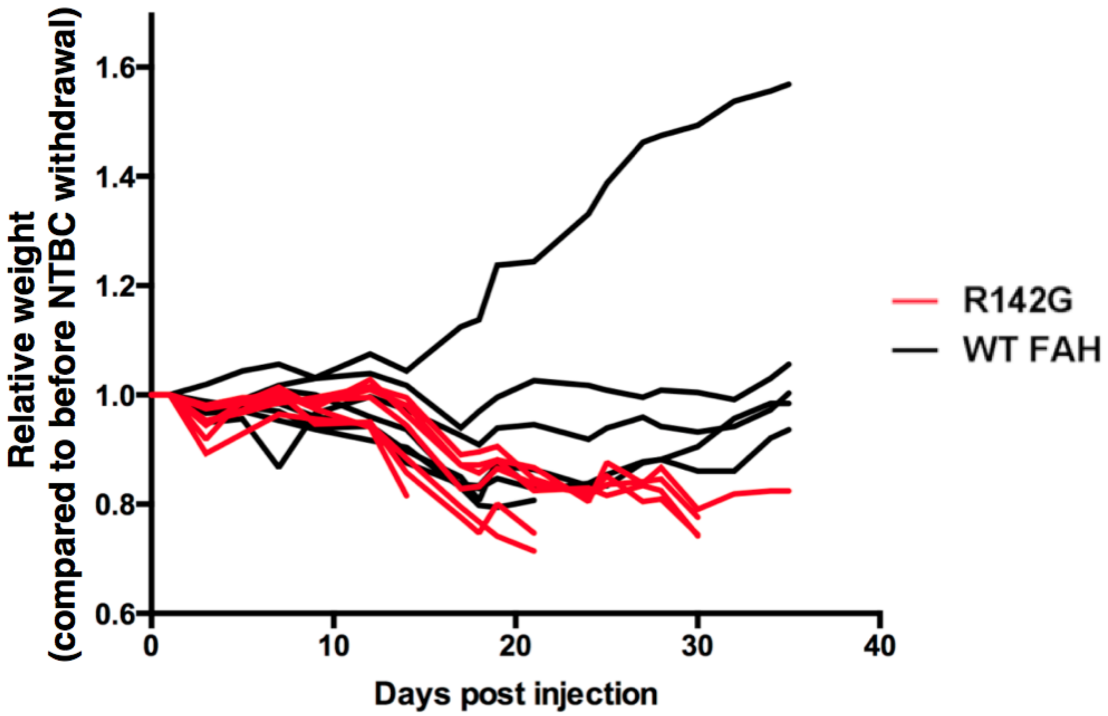

Supp. Figure S4. Relative weight (compared to before NTBC withdrawl) for the second series of mice.

Graph showing the relative weight (compared to before NTBC withdrawl) in Fah-/- mice over time. A second series of Fah-/- mice received hydrodynamic tail vein injections with either pKT2/hFAHIL or pKT2/R142G/hFAHIL in addition to pKUb-SB100X as in the previous experiment. When mice surpassed the >20% weight loss threshold they were euthanized rather than being placed back on 2-(2-nitro-4-trifluoro-methylbenzyol)-1,3 cyclo-hexanedione. The weights of the mice followed a similar trend to the mice in the first experiment. Mice expressing the wild-type fumarylacetoacetate hydrolase had a slight decrease in initial weight, but they recovered or exceed their day 0 weights by the end of the study (day 35), indicating therapeutic correction of fumarylacetoacetate hydrolase deficiency. Mice expressing mutant FAH lost weight more slowly, but all had lost >20% total weight by day 30 with no evidence of recovery.

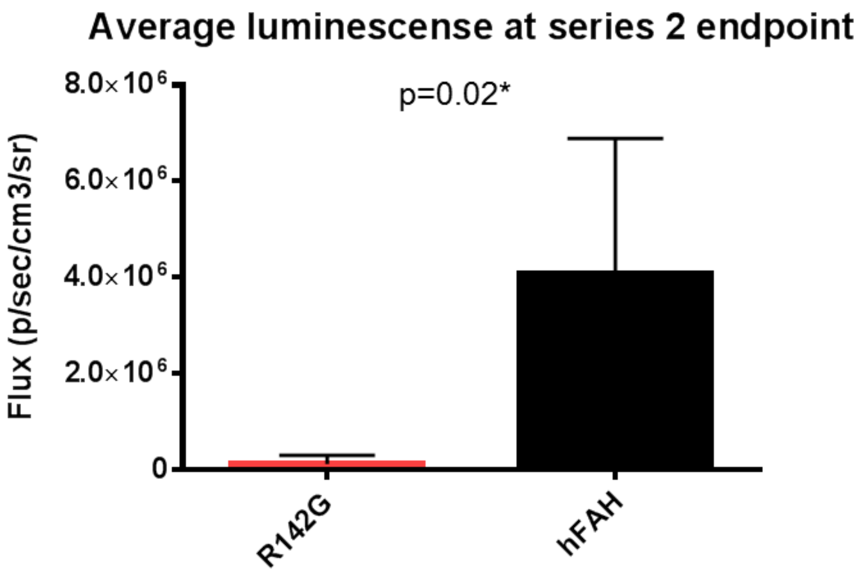

<u>Supp. Figure S5. Average luminescence at the time of tissue harvest for the second series of mice.</u>

Luminescence intensity in mice expressing wild type or p.R142G mutant fumarylacetoacetate hydrolase at the study endpoint (second series of mice). A significant difference was detected between these two groups, indicating correction of the fumarylacetoacetate hydrolase deficiency in mice expressing the wild-type form but not the mutant form.

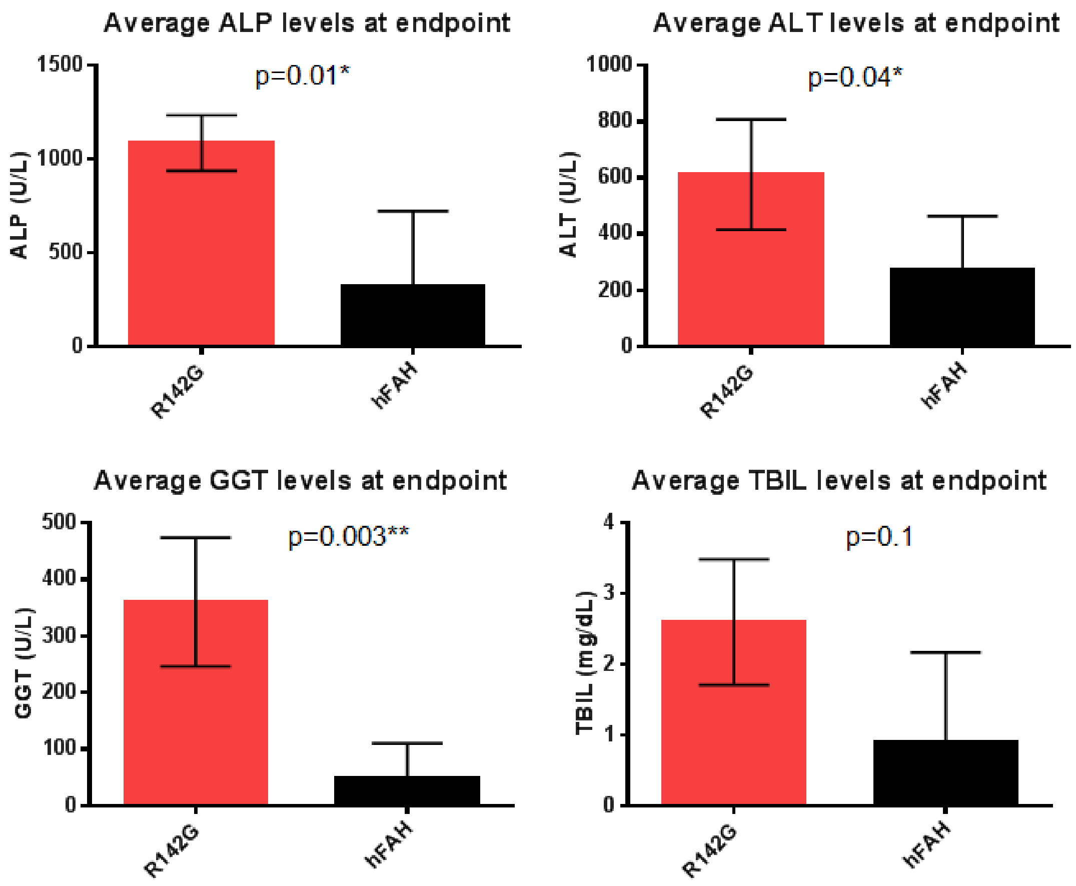

Supp. Figure S6. Liver panel values for all mice at the time of tissue harvest.

Alanine aminotransferase, alkaline phosphatase, and gamma glutamyl transferase levels were significantly elevated in mice expressing mutant fumarylacetoacetate hydrolase compared with mice expressing wild-type fumarylacetoacetate hydrolase at the study endpoint. A nonsignificant difference in total bilirubin was seen between these two groups.
